# Intercostal Nerve Block for Bilateral Thoracoscopic Sympathectomy: A Prospective Observational Cohort Comparison of Three Analgesic Protocols

**DOI:** 10.3390/jcm15145755

**Published:** 2026-07-22

**Authors:** Marko Kantar, Ivan Kuhajda, Bojan Joksimović, Radovan Jurić, Nemanja Lazendić, Danijela Radulović

**Affiliations:** 1 Clinic for Thoracic Surgery, University Clinical Center of the Republic of Srpska, 78000 Banja Luka, Bosnia and Herzegovina; kantarmarko@yahoo.com (M.K.); rjuric1996@gmail.com (R.J.); 2 Faculty of Medicine, University of Banja Luka, 78000 Banja Luka, Bosnia and Herzegovina; nemanjalazendic@yahoo.com; 3 Clinic for Thoracic Surgery, Institute for Pulmonary Diseases of Vojvodina, 21204 Sremska Kamenica, Serbia; 4 Department of Surgery, Faculty of Medicine, University of Novi Sad, 21000 Novi Sad, Serbia; 5 Faculty of Medicine Foča, University of East Sarajevo, 73300 Foča, Bosnia and Herzegovina; bojannjoksimovic@gmail.com; 6 Department for Rehabilitation of Patients with Chronic Non-Communicable Diseases, Institute for Physical Medicine and Rehabilitation and Orthopedic Surgery “Dr Miroslav Zotović”, 78000 Banja Luka, Bosnia and Herzegovina

**Keywords:** primary focal hyperhidrosis, bilateral thoracoscopic sympathectomy, intercostal nerve block, postoperative pain, video-assisted thoracoscopic surgery, opioid-sparing analgesia

## Abstract

**Background/Objectives:** Bilateral thoracoscopic sympathectomy (BTS) is the definitive treatment for primary focal hyperhidrosis, yet postoperative pain remains an undertreated consequence with no established analgesic consensus. This study compared three analgesic regimens on pain trajectory, opioid consumption, and functional recovery following BTS. **Methods:** In this prospective observational cohort study, 300 patients undergoing BTS were allocated in a non-randomized manner reflecting institutional practice to three parallel cohorts (*n* = 100 each): Group 1 received systemic opioids alone; Group 2 received opioids plus pre-emptive local anesthetic wound infiltration; and Group 3 received opioids plus thoracoscopic-guided intercostal nerve block (ICNB). Primary outcomes included pain intensity on the Numerical Rating Scale and total opioid consumption. **Results:** ICNB patients reported significantly lower mean peak pain scores (3.8 ± 1.7) versus local infiltration (4.6 ± 1.9) and opioid-only groups (5.2 ± 2.1; *p* < 0.001). Total opioid consumption was reduced by 38% in the ICNB group (12.4 ± 4.8 mg vs. 20.1 ± 6.3 mg; *p* < 0.001). Moderate-to-severe pain occurred in 42.0% of ICNB patients versus 74.0% of controls (*p* < 0.001). ICNB was independently associated with reduced moderate-to-severe pain (adjusted odds ratio (AOR) 0.31; 95% confidence interval (CI) 0.17–0.56; *p* < 0.001). ICNB patients returned to normal activities faster (median 3 vs. 5 days; *p* = 0.008) with higher satisfaction (91.0% vs. 78.0%; *p* = 0.044). **Conclusions:** In this observational cohort, ICNB was associated with significantly lower postoperative pain scores, reduced opioid consumption, and faster functional recovery following BTS. These findings suggest that ICNB may be an effective adjunct analgesic strategy for ambulatory BTS, pending confirmation in randomized controlled trials.

## 1. Introduction

Primary focal hyperhidrosis (PFH) is a disorder characterized by excessive, uncontrollable sweating that surpasses the body’s physiological requirements for thermoregulation [1]. Although it is not a life-threatening condition, its prevalence—estimated at approximately 2.8% of the global population—means it affects a substantial number of individuals, typically manifesting in childhood or adolescence with peak incidence in the second and third decades of life [2,3]. The condition predominantly affects the palms, axillae, soles, and face, either in isolation or in various combinations, and carries a well-documented autosomal dominant inheritance pattern with variable penetrance [4]. PFH is more than a cosmetic concern; it has a real impact across multiple domains of daily living. Patients frequently report profound functional impairments, such as difficulty handling paper or electronic equipment, alongside significant psychosocial distress including social withdrawal, diminished self-esteem, and vocational limitations [5,6,7,8]. Consequently, the baseline quality of life (QoL) reported by individuals with severe PFH is often comparable to that seen in chronic debilitating diseases, with over 60% of surgical candidates rating their preoperative QoL as poor or extremely poor [9,10].

The therapeutic algorithm for PFH encompasses a range of conservative and interventional options. Topical aluminum chloride preparations, iontophoresis, systemic anticholinergics such as oxybutynin, and intradermal botulinum toxin injections all offer temporary symptomatic relief, yet their long-term utility is often constrained by limited durability, local adverse effects, prohibitive cost, or patient inconvenience [11,12]. For patients seeking a definitive and permanent resolution, bilateral thoracoscopic sympathectomy (BTS) has emerged as the gold-standard surgical intervention over the past three decades [13]. The procedure involves thoracoscopic transection of the sympathetic chain at specific thoracic levels—guided by the localization of the hyperhidrosis—and has been consistently shown to achieve cure rates exceeding 95% for palmar disease with minimal perioperative morbidity and mortality [14,15]. Importantly, recent longitudinal cohort studies have confirmed that nearly 90% of patients remain satisfied with their outcome up to a decade after surgery [16].

Despite its undisputed efficacy, BTS is not without attendant drawbacks, the most significant of which is the development of compensatory hyperhidrosis (CH)—a phenomenon defined by the onset of new, often distressing sweating in previously unaffected areas, most commonly the trunk, back, and thighs [17,18]. Reported CH incidence varies widely (approximately 50% to over 90%), largely reflecting differences in surgical technique, the level of sympathetic chain interruption, and inconsistent severity definitions [19,20]. Extensive clinical research has established that the risk of developing severe CH is modifiable and primarily governed by the extent and cranial level of the sympathectomy [21,22,23]. This understanding has driven a progressive evolution in surgical practice toward more limited and caudally targeted sympathicotomies, such as isolated R3 or R4 interruption for palmar disease, which achieve an optimal balance between symptom resolution and minimization of adverse effects [23,24].

In contrast to this extensive literature on optimizing surgical levels to mitigate CH, the postoperative pain experience following BTS, and the question of how best to control it, has received comparatively little systematic investigation; this analgesic gap is the focus of the present study. Acute postoperative pain is a near-universal occurrence, reported by 80–98% of patients in available series, and is understood to be multifactorial in origin, arising from the chest wall incisions [25,26], trocar-related trauma to the intercostal neurovascular bundles, and the thermal or mechanical injury to the parietal pleura and periosteum during the sympathectomy itself [27,28]. Although the pain is generally of moderate intensity and self-limiting—typically resolving within two weeks—it nonetheless represents a significant source of early postoperative discomfort and has the potential to impede immediate recovery, delay hospital discharge, and compromise patient satisfaction during the critical perioperative period [29,30]. Various analgesic strategies have been described for thoracoscopic procedures, ranging from systemic opioids and non-steroidal anti-inflammatory drugs (NSAIDs) to regional techniques such as intercostal nerve blocks (ICNB), paravertebral blocks, and intrapleural or pre-emptive local anesthetic (LA) infiltration at the port sites [31,32].

Despite the widespread acknowledgment of post-sympathectomy pain, there remains a clear gap in evidence regarding the comparative effectiveness of these different analgesic modalities in the specific context of BTS performed as an ambulatory or short-stay procedure. The existing literature is characterized by small, heterogeneous studies, a paucity of head-to-head comparisons, and an absence of a standardized, evidence-based analgesic protocol [33]. Even recent trials [32,34] have not definitively established the optimal approach. Moreover, the interplay between analgesic efficacy, opioid consumption, and the patient’s ability to meet fast-track discharge criteria has not been rigorously defined in this patient population.

Therefore, the primary objective of this prospective, controlled, three-group observational study was to address this unmet clinical need by systematically comparing the efficacy of three distinct perioperative analgesic techniques—systemic opioids alone, systemic opioids supplemented with pre-emptive LA wound infiltration, and systemic opioids supplemented with ICNB—in patients undergoing BTS for PFH. Our analysis focused on a comprehensive panel of outcomes including pain trajectory over the first 24 h, total opioid consumption expressed as morphine milligram equivalents, the incidence and severity of adverse effects, time to return to normal daily activities, and patient satisfaction. We hypothesized that the addition of a regional anesthetic technique, specifically ICNB, would confer superior pain control, facilitate opioid-sparing, and accelerate functional recovery without increasing the risk of perioperative complications, thereby providing a clear evidence-based framework to optimize the postoperative experience for this patient cohort.

## 2. Materials and Methods

### 2.1. Study Design and Ethical Oversight

This study is reported in accordance with the STROBE (Strengthening the Reporting of Observational studies in Epidemiology) guidelines for observational studies [35] (Supplementary File S1—filled checklist). The work we report here was carried out as a prospective observational cohort study in which we compared three different approaches to perioperative pain control in patients scheduled for bilateral thoracoscopic sympathectomy. The protocol was reviewed in advance and received approval from the Institutional Ethics Committee of the Institute for Pulmonary Diseases of Vojvodina (approval number: 85-I/9, dated 6 February 2018). Every aspect of the study was conducted in accordance with the principles laid out in the Declaration of Helsinki. Before any study-related procedures were initiated, all participants received a detailed explanation of the surgical procedure and the three analgesic protocols in routine use at our institution, and were informed that participation was entirely voluntary. Written informed consent was obtained from every individual who agreed to take part.

### 2.2. Patient Selection and Eligibility Criteria

We screened consecutive adult patients who carried a confirmed diagnosis of primary focal hyperhidrosis and who were scheduled to undergo elective bilateral thoracoscopic sympathectomy at the Clinic for Thoracic Surgery, Institute for Pulmonary Diseases of Vojvodina. The enrollment window extended from 15 February 2018 to 31 December 2020. To be considered for inclusion, individuals needed to be between the ages of 18 and 50 years and to have an American Society of Anesthesiologists physical status classification of I or II. In terms of prior treatment, patients were eligible if they had either tried and not achieved satisfactory results with conservative measures—such as prescription antiperspirants, iontophoresis, oral anticholinergics, or botulinum toxin—or if they had declined further conservative management in favor of a definitive surgical solution.

We applied several exclusion criteria to ensure that the postoperative pain experience would reflect the surgical procedure and the analgesic intervention rather than pre-existing conditions or anatomical abnormalities. Individuals were excluded if they had ever undergone any sort of operation on the thoracic cavity, whether a formal thoracotomy, a prior video-assisted thoracoscopic procedure for any indication, or even a previous chest tube placement. A history of rib fractures, a diagnosis of a chronic pain syndrome requiring daily analgesic medication regardless of its underlying cause, or a history of massive pneumonia or pleural empyema that might have resulted in pleural adhesions were also grounds for exclusion. Patients receiving chronic opioid, anticonvulsant, antidepressant, or other psychotropic medication with potential analgesic activity were not enrolled, so as to avoid confounding of postoperative pain assessment; no enrolled patient was receiving such therapy at baseline. We further excluded patients with significant impairment of cardiac or pulmonary function that would make even brief periods of single-lung ventilation unsafe, those with known disorders of coagulation or bleeding, and anyone with a documented allergy to amide-type local anesthetics or to any of the opioid medications specified in the protocol. A body mass index greater than 35 kg/m^2^ was an exclusion criterion, as was any psychiatric or cognitive condition that might interfere with a patient’s ability to understand the Numerical Rating Scale (NRS) or to reliably complete the study questionnaires. Pregnant and breastfeeding women were not enrolled.

**Figure 1:**
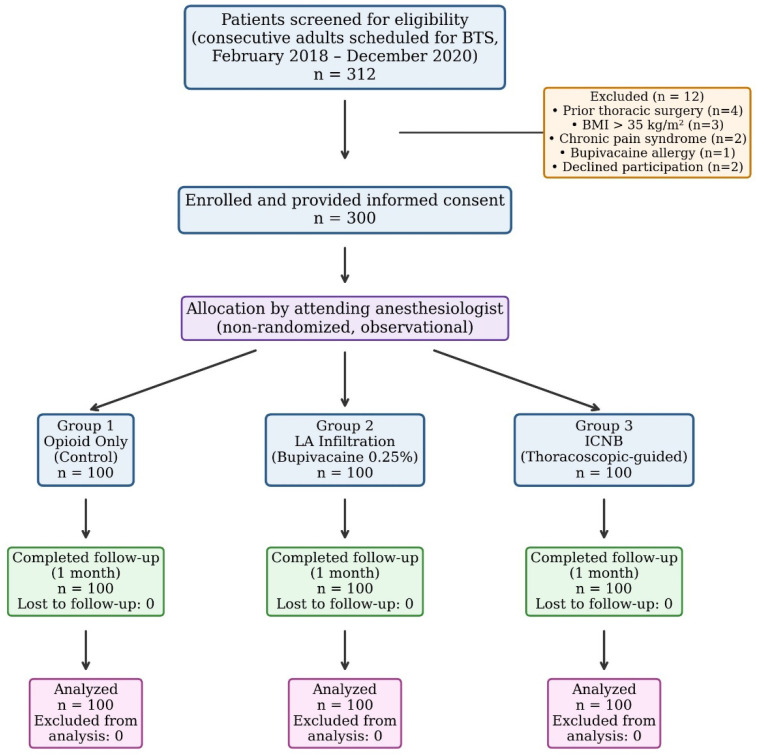
STROBE flow diagram of study participants. Of 312 consecutive adults screened between February 2018 and December 2020, 12 were excluded for the reasons listed. The remaining 300 patients were enrolled and assigned by the attending anesthesiologist to one of three perioperative analgesic protocols (*n* = 100 per group). All participants completed 1-month follow-up and were included in the final analysis. Group allocation was non-randomized and reflected institutional practice patterns. BTS, bilateral thoracoscopic sympathectomy; LA, local anesthetic; ICNB, intercostal nerve block.

The flow of participants through screening, eligibility, allocation, and follow-up is summarized in Figure 1.

### 2.3. Study Groups and Analgesic Protocols

Patients who met all eligibility criteria and agreed to participate were assigned to one of three parallel cohorts according to the perioperative analgesic protocol that was active at the time of their operation. The decision regarding which protocol to use was made by the attending anesthesiologist in discussion with the operating surgeon. Allocation reflected institutional practice patterns rather than randomization: the choice was guided by the anesthesiologist’s familiarity with the regional technique, scheduled operating-room time, and—importantly—the gradual institutional uptake of ICNB during the study period. To address concerns about temporal confounding, the distribution of protocols by enrollment year is presented as Supplementary Data; all three protocols were used in each calendar year of the study, although the proportion of ICNB cases increased over time. Distribution of group allocation by enrollment month is shown in Supplementary Table S1.

The three cohorts were defined by the following analgesic regimens.

Group 1: Systemic Opioid Analgesia Alone

This cohort served as our reference or control group. These patients received systemic opioids according to a standardized regimen and did not receive any form of regional anesthesia or local wound infiltration. The opioid component consisted of intravenous fentanyl given at induction at a dose of 3 μg per kilogram of actual body weight. Additional intraoperative boluses could be administered at the discretion of the anesthesiologist if needed to maintain hemodynamic stability. At the conclusion of the operation, patients received a single subcutaneous injection of morphine sulfate at a dose of 5 mg. Of note, multimodal non-opioid baseline analgesia (paracetamol, Non-steroidal Anti-inflammatory Drugs, NSAIDs) was not routinely administered as part of any of the three protocols during the study period; this reflects institutional practice at the time of the study.

Group 2: Preemptive Local Anesthetic Infiltration

Individuals in this cohort received the identical systemic opioid regimen described for Group 1. In addition, they underwent preemptive infiltration of the surgical port sites with a local anesthetic solution. After the patient was anesthetized and the operative field had been prepped and draped, but before the surgeon made the first skin incision, each of the two planned 5 mm port sites on each side of the chest was infiltrated with 2 mL of 0.25% bupivacaine hydrochloride. This amounted to 4 mL per hemithorax (2 mL × 2 ports) and 8 mL total per patient (across both hemithoraces), corresponding to a total bupivacaine dose of 20 mg. The infiltrations were carried out through the thoracoscope to make certain that the anesthetic was deposited accurately into the subcutaneous tissue and the superficial intercostal muscle layers, steering clear of any vessels or the pleural space itself.

Group 3: Intercostal Nerve Block

Patients in the third cohort received the same systemic opioid protocol as the other two groups, but their regional analgesic supplement took the form of an intercostal nerve block performed with thoracoscopic guidance. Once the ports had been placed, the scope introduced, and the sympathetic chain clearly identified, but before any transection of the chain was undertaken, we performed the block. Using a long 22-gauge spinal needle that was advanced percutaneously under direct thoracoscopic observation to verify its position, we injected 2 mL of 0.25% bupivacaine into the intercostal space at the vertebral levels corresponding to the surgical port sites, depositing the anesthetic beneath the parietal pleura adjacent to the intercostal neurovascular bundle. Because the injection was performed thoracoscopically at the angle of the rib near the paravertebral gutter, the technique is most accurately described as a thoracoscopic-guided proximal intercostal nerve block; we acknowledge that, depending on the precise spread of local anesthetic at this medial location, the block may share features of a paravertebral-type field block. The aim was to anesthetize the intercostal nerves carrying sensory input from the operative field and the neighboring chest wall. The identical technique and dosing were applied to both sides of the chest.

### 2.4. Standardized Anesthetic and Surgical Technique

To keep variation in postoperative pain that might stem from differences in surgical technique or anesthetic depth to an absolute minimum, we adhered to a uniform operative and anesthetic protocol for every patient in the study.

Anesthetic Management

General anesthesia was used in all cases, with endotracheal intubation accomplished using a standard single-lumen tube. Induction was achieved with intravenous propofol at 2 mg per kilogram of actual body weight. Anesthesia was maintained with sevoflurane vaporized in a mixture of oxygen and air, with the delivered concentration adjusted according to clinical signs and hemodynamic parameters to maintain an adequate depth of anesthesia. Muscle relaxation to facilitate intubation and to provide optimal operating conditions during the period of lung collapse was obtained with rocuronium bromide given at a dose of 0.6 mg per kilogram, with supplemental doses administered as needed. Routine intraoperative monitoring, consistent with American Society of Anesthesiologists guidelines, included continuous three-lead electrocardiography, non-invasive blood pressure readings taken every five minutes, continuous pulse oximetry, and capnography. For lung collapse, the endotracheal tube was briefly disconnected from the ventilator circuit, allowing passive exhalation and collapse of the operative hemithorax to create the working space required for the thoracoscopic portion of the case.

Surgical Technique

The surgical procedure itself was carried out according to a standardized institutional protocol. Patients were placed in a semi-Fowler position, with the upper body elevated to about 45 degrees from the horizontal and both arms abducted at the shoulder to roughly 90 degrees. We have previously reported that this positioning arrangement shortens operative times and improves access for the surgeon. The skin of the entire anterior and lateral chest wall was prepped with povidone-iodine solution and draped in a sterile fashion that permitted access to both sides of the chest without having to reposition the patient. On each side, two 5 mm incisions were made. The first incision was placed in the third intercostal space along the anterior axillary line, and the second was made in the fourth intercostal space along the mid-axillary line. A 5 mm trocar was inserted through each incision. A 5 mm rigid thoracoscope with a 30-degree angled lens was introduced through one port, allowing us to inspect the pleural cavity and identify the sympathetic chain where it runs longitudinally across the necks of the ribs. An endoscopic ultrasonic hook device was then brought in through the other port.

Under direct vision, we transected the sympathetic chain and any identifiable Kuntz fibers. The exact level of transection was tailored to the patient’s individual pattern of hyperhidrosis. For those with predominantly palmar symptoms, we divided the chain at the level of the second and third thoracic ganglia. For axillary-predominant disease, we targeted the third and fourth thoracic ganglia. When both distributions were involved, the transection spanned the second through fourth thoracic levels. After confirming that hemostasis was satisfactory, the anesthesiologist applied positive airway pressure to re-expand the lung while we watched through the scope. A small chest tube was momentarily passed through one of the port sites and connected to suction to evacuate any residual intrapleural air, and then it was promptly removed as the anesthesiologist maintained positive pressure. The skin incisions were closed with absorbable subcuticular sutures and reinforced with sterile adhesive strips. No chest drains remained in place at the end of the procedure.

### 2.5. Data Collection and Outcome Measures

Data were gathered prospectively from two main sources: abstraction from the medical record and a series of standardized questionnaires that patients completed at specified time points. The nursing staff and other personnel who recorded pain scores and collected follow-up information were not informed which analgesic protocol any given patient had received.

Baseline Demographic and Clinical Characteristics

From the preoperative evaluation and the medical chart, we abstracted the following information: age, gender, body mass index (weight in kilograms divided by the square of height in meters), the number and specific anatomical locations of body regions affected by hyperhidrosis (categorized as palmar, axillary, facial, or plantar), whether the patient had previously attempted conservative therapy and which specific modalities had been tried, and whether there was a family history of hyperhidrosis in a first-degree relative. To minimize observer bias, postoperative pain scores were recorded by post-anesthesia care unit nursing staff using standardized assessment forms. However, complete blinding was not feasible given that the operative record documented the regional technique, and we acknowledge this as a limitation. Data abstraction and outcome adjudication were performed by a research team member who was not involved in intraoperative care.

Preoperative Disease Severity and Quality of Life Assessment

Before the operation, each patient completed the Hyperhidrosis Disease Severity Scale, a validated four-point instrument that captures how tolerable the sweating is and the extent to which it disrupts everyday life. A score of 1 means the sweating is never noticeable and never gets in the way; a 2 means it is tolerable but does sometimes interfere; a 3 means it is barely tolerable and frequently interferes; and a 4 means it is intolerable and constantly interferes. For the purposes of analysis, we grouped grades 1 and 2 together as mild-to-moderate disease and grades 3 and 4 as severe disease. Patients also gave a global rating of their disease-specific quality of life on a four-point scale anchored as follows: 0, extremely poor; 1, poor; 2, good; and 3, excellent.

Postoperative Pain Assessment: Intensity, Trajectory, and Localization

Our primary focus was on postoperative pain intensity, which we measured using the 11-point Numerical Rating Scale. On this scale, a score of 0 corresponds to “no pain at all” and a score of 10 corresponds to “the worst pain you can imagine.” Pain ratings were obtained at four standard times: upon emergence from anesthesia, defined as the moment the patient could follow a simple command, and then at 6, 12, and 24 h after extubation. The nurses in the post-anesthesia care unit who documented these scores were unaware of the analgesic protocol the patient had been assigned to. We also noted the highest pain score recorded during the entire observation period. For some of the analyses, we collapsed pain intensity into categories: mild pain was an NRS of 1 to 3, moderate pain was 4 to 6, and severe pain was 7 to 10. We defined moderate-to-severe pain as an NRS of 4 or above.

In addition to the intensity ratings, we asked patients at their follow-up appointment to tell us how long they had experienced any discomfort that they felt was related to the surgery. They chose from a set of categorical responses: less than a week, one to two weeks, two weeks to a month, or longer than a month.

To understand where the pain was located, we showed patients a simple body diagram and asked them to point out the areas of discomfort. The responses were later coded by the research team into one of several categories: surgical port sites, anterior chest wall or retrosternal area, posterior thorax or interscapular region, right hemithorax, or left hemithorax.

Analgesic Consumption and Opioid Requirements

We tallied all the opioids administered during the operation and over the subsequent 24 h and converted these amounts to intravenous morphine milligram equivalents using standard conversion factors [36]: 1 microgram of fentanyl given intravenously was taken as equivalent to 0.1 mg of intravenous morphine, and subcutaneous morphine was considered to be equivalent to intravenous morphine on a milligram-for-milligram basis. We considered rescue analgesia to be any pain medication given above and beyond the standardized protocol, whether it was requested by the patient or initiated by the nursing staff for breakthrough pain. We recorded the specific drug used for rescue and classified it as an NSAID (ibuprofen or diclofenac according to our hospital formulary), acetaminophen, or a combination of the two. We also noted the time that elapsed before the first dose of rescue medication was needed.

Assessment of Compensatory Hyperhidrosis

When patients returned for follow-up, we specifically asked whether they had noticed new sweating or a worsening of sweating in parts of the body that had not been problematic before the operation. Those who answered affirmatively were asked to characterize the severity of this compensatory sweating using a three-level scheme: mild, meaning it was noticeable but not really bothersome; moderate, meaning it was bothersome but did not prevent them from doing their usual activities; or severe, meaning it interfered with daily life or caused considerable distress. We also inquired about gustatory sweating, which is sweating of the face or scalp triggered by eating certain foods.

Functional Recovery and Patient-Reported Outcomes

To gauge the speed of recovery, we posed the following question at the follow-up visit: “How many days after your surgery did it take before you felt you were back to your usual routine, including work, household chores, or social activities?” The answer was recorded simply as a number of days.

We readministered the Hyperhidrosis Disease Severity Scale (HDSS) at the follow-up appointment. We considered a drop of two or more points from the preoperative score to represent a clinically meaningful improvement. Patients also provided a follow-up global quality of life rating using the same four-point scale they had seen before surgery.

We assessed patient satisfaction with a 5-point Likert scale that offered the following choices: very dissatisfied, dissatisfied, neutral, satisfied, and very satisfied. When we prepared the data for presentation, we collapsed these into three categories: very satisfied, satisfied, and a combined neutral/dissatisfied group. We also asked a straightforward yes-or-no question: “Knowing what you know now, would you recommend this operation to a friend or family member who had the same problem with sweating?”

Safety and Perioperative Adverse Events

Any adverse events that occurred during the operation or in the immediate recovery period were documented in the medical record and subsequently extracted by the research team. We kept a particular eye out for bradycardia that required treatment, transient drops in oxygen saturation, nausea and vomiting, itching, lightheadedness, transient tingling or numbness in the arms, and shortness of breath. We were especially attentive to any complication that might be specifically related to the intercostal block, such as a pneumothorax that needed intervention or any sign of systemic toxicity from the local anesthetic. Finally, we recorded the total time the patient spent in the post-anesthesia care unit and in the hospital, measured in hours from the moment of extubation until discharge criteria were satisfied.

### 2.6. Statistical Analysis

The statistical work for this study was performed using IBM SPSS Statistics, Version 26.0, with mixed-effects analyses conducted in R (version 4.3.0) using the lme4 package. Before we ran any comparative tests, we looked at the distribution of each continuous variable using the Shapiro–Wilk test and by examining Q-Q plots. Data that followed a normal distribution are reported as means with their standard deviations. Variables that were not normally distributed are summarized as medians with interquartile ranges. Categorical data are presented as counts and percentages. There were no missing data for the primary outcomes; for the small number of secondary variables with incomplete responses (pain-duration category, reported by 99, 98, and 98 patients in Groups 1–3, respectively), analyses were performed on complete cases, and the relevant denominators are stated in the table footnotes.

Before applying parametric tests, the assumptions of residual normality and homogeneity of variance were formally assessed using the Shapiro–Wilk test and Levene’s test, respectively, supplemented by visual inspection of Q-Q and residual-versus-fitted plots; for the mixed-effects models, residual normality and homoscedasticity were verified by inspection of residual plots. Where these assumptions were satisfied, normally distributed continuous measures were compared across the three groups using One-Way Analysis of Variance. If the overall F-test came back with a *p*-value below 0.05, we then conducted post hoc pairwise comparisons using the Tukey Honestly Significant Difference (HSD) test. For data that did not meet the assumption of normality, we relied on the Kruskal–Wallis test to compare the distributions across the three cohorts. Categorical outcomes were compared with the Chi-square test. For ordered categorical variables, we reported the Chi-square *p*-value alongside the linear-by-linear association (Cochran–Armitage) trend test, which is more sensitive to monotonic trends across ordered categories. For within-patient pre- to postoperative changes, we used the Wilcoxon signed-rank test.

To analyze the longitudinal trajectory of postoperative pain across the four assessment time points (emergence, 6 h, 12 h, 24 h), we constructed a linear mixed-effects model with NRS score as the dependent variable; analgesic group, time, and the group × time interaction as fixed effects; and patient as a random intercept to account for within-subject correlation arising from repeated measurements on the same individual. Pairwise comparisons between analgesic groups at each time point were derived from the fitted model using estimated marginal means (EMMs) with Tukey adjustment to control the family-wise error rate.

To address possible secular changes during enrollment (February 2018–December 2020), we re-analyzed peak NRS using a linear mixed-effects model with enrollment year as a categorical fixed effect, and re-examined the proportion with moderate-to-severe pain within each year stratum by Chi-square test. Findings were considered robust if between-group differences were preserved across 2018, 2019, and 2020.

We also built a multivariable logistic regression model to see which factors were independently associated with the likelihood of experiencing moderate-to-severe pain, defined as an NRS score of 4 or higher. We entered variables into the model if they showed a *p*-value below 0.10 in the univariable screen, or if we felt they were important to include based on what has been published in the literature, regardless of their univariable association. The final set of covariates included age, gender, body mass index, the number of affected body regions, the baseline HDSS severity category, and the assigned analgesic group. Multicollinearity among covariates was assessed using variance inflation factors (VIF); all values were below 2.0, indicating no problematic collinearity. With 177 moderate-to-severe pain events and six covariates entered, the model satisfied the conventional minimum of at least 10 events per variable, mitigating the risk of overfitting. The results of this model are expressed as adjusted odds ratios (AOR) accompanied by 95% confidence intervals (CI). To evaluate how well the model fit the data, we used the Hosmer–Lemeshow goodness-of-fit test. A non-significant *p*-value on this test suggests that the model’s predictions and the actual outcomes are in reasonable agreement. We assessed the model’s ability to discriminate between patients who did and did not have moderate-to-severe pain by computing the area under the receiver operating characteristic curve. For the primary between-group comparisons, effect sizes are reported as Cohen’s d for pairwise mean differences and as eta-squared for the omnibus ANOVA, each accompanied by 95% confidence intervals; for categorical outcomes, effect sizes are expressed as odds ratios or relative risks with 95% confidence intervals. For omnibus group comparisons, the F statistic is reported together with its degrees of freedom in the form F(df1, df2), and for categorical outcomes the strength of association is additionally summarized using Cramer’s V; eta-squared is interpreted with values of approximately 0.01, 0.06, and 0.14 denoting small, medium, and large effects, respectively. All *p*-values reported in this paper are two-sided, and we considered a value of less than 0.05 to indicate statistical significance. For non-parametric between-group comparisons (Kruskal–Wallis test), the H statistic is reported with its degrees of freedom and epsilon-squared (ε^2^) as the corresponding effect size. To address the issue of multiple comparisons across secondary and exploratory endpoints, we applied a Benjamini–Hochberg false discovery rate (FDR) correction across the family of 15 secondary outcome *p*-values (Supplementary Table S2).

### 2.7. Sample Size Considerations

The sample size was calculated a priori using G*Power version 3.1.9.7. Drawing on earlier data from our own institution as well as figures reported in the published literature on thoracoscopic sympathectomy, we estimated that enrolling 100 patients per cohort would give us more than 80% power to detect a difference of 1.5 points on the 11-point NRS for peak postoperative pain (corresponding to Cohen’s f ≈ 0.20, a medium effect size). The 1.5-point threshold was chosen as the minimal clinically important difference (MCID) for postoperative pain based on published literature [37]. This calculation assumed a standard deviation of about 2.0 points, a two-sided alpha of 0.05, and a roughly 10% rate of incomplete data or loss to follow-up. The sample-size requirement was derived for the primary three-group ANOVA of peak NRS and represented the most demanding among the planned analyses; secondary and multivariable analyses were not separately powered and are reported as exploratory. We also felt that a sample of this size would allow us to estimate our secondary outcomes—such as the proportion of patients achieving a substantial improvement in HDSS grade or the incidence of compensatory hyperhidrosis—with acceptable precision.

## 3. Results

### 3.1. Baseline Characteristics of the Study Cohort

The final analysis included 300 patients who underwent bilateral thoracoscopic sympathectomy, with 100 individuals assigned to each of the three study groups. The three cohorts were well balanced with respect to demographic and clinical characteristics, with no statistically significant differences in age, sex, body mass index, distribution of affected body regions, baseline HDSS severity, prior conservative therapy, family history, or preoperative quality of life (all *p* > 0.05; Table 1).

### 3.2. Postoperative Pain Profile and Opioid-Sparing Effects

While some degree of postoperative discomfort was reported almost universally across the cohort, the intensity and trajectory of pain differed significantly based on the analgesic strategy employed. In the linear mixed-effects model of NRS over the four time points, there was a significant main effect of group (*p* < 0.001), a significant main effect of time (*p* < 0.001), and a significant group × time interaction (*p* < 0.001), indicating that the difference between groups varied across the postoperative period and was most pronounced during the first 12 h. Patients in the control group, receiving only systemic opioids, reported a mean peak NRS pain score of 5.2, squarely in the moderate-to-severe range. This contrasted with a mean peak score of 4.6 in the local anesthetic infiltration group and a notably lower mean of 3.8 in the intercostal nerve block group (One-Way ANOVA F(2, 297) = 24.6, *p* < 0.001; eta-squared = 0.14, 95% CI 0.08–0.21). Post hoc multiple comparisons clarified that the advantage of the intercostal block was robust; it was superior not only to the opioid-only approach (mean difference −1.4, 95% CI −1.9 to −0.9; Cohen’s d = 0.72, 95% CI 0.43–1.01; *p* < 0.001) but also to simple port-site infiltration (mean difference −0.8, 95% CI −1.4 to −0.2; Cohen’s d = 0.41, 95% CI 0.13–0.69; *p* = 0.008). Even local infiltration alone offered a modest but statistically detectable benefit over the control condition (mean difference −0.6, 95% CI −1.1 to −0.1; Cohen’s d = 0.30, 95% CI 0.02–0.58; *p* = 0.023) (Table 2).

**Figure 2:**
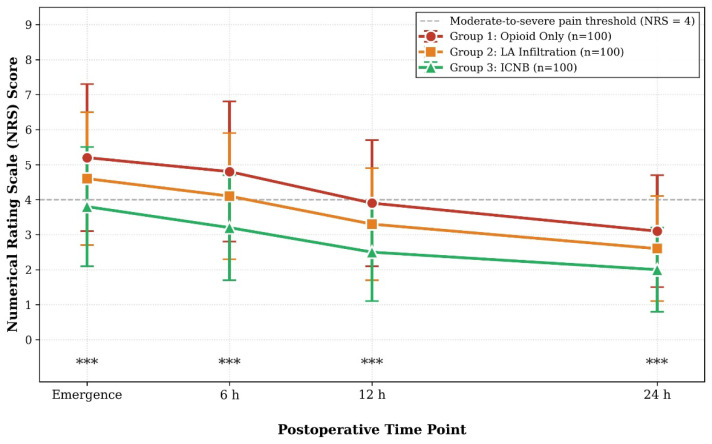
Postoperative pain trajectory by analgesic group. Mean Numerical Rating Scale (NRS) scores ± standard deviation at four postoperative time points (emergence from anesthesia, 6 h, 12 h, and 24 h after extubation) for each of the three analgesic cohorts. The horizontal dashed line at NRS = 4 indicates the threshold for moderate-to-severe pain. The data were analyzed using a linear mixed-effects model with group, time, and group × time interaction as fixed effects and patient as a random intercept; the model demonstrated significant main effects of group (*p* < 0.001), time (*p* < 0.001), and a significant group × time interaction (*p* < 0.001), confirming that the magnitude of the between-group difference varied across the postoperative period and was most pronounced during the first 12 h. *** *p* < 0.001 for pairwise between-group comparisons at each time point, derived from estimated marginal means with Tukey adjustment. LA, local anesthetic; ICNB, intercostal nerve block.

The pattern of pain over time showed that the analgesic effect of the intercostal block was sustained (Figure 2). At the 6 h postoperative mark, the mean NRS score in the ICNB group was just 3.2, compared to 4.1 in the local infiltration group and 4.8 in the control group (*p* < 0.001). By the 12 h mark, the ICNB cohort continued to report lower pain scores (2.5 vs. 3.3 and 3.9, respectively), and this pattern persisted, albeit with diminishing absolute differences, through the first full postoperative day. Better pain control also meant fewer patients needed supplemental analgesia. While two-thirds of patients in the control group required rescue analgesics, that proportion fell to 57% in the local infiltration group and further declined to 42% in the ICNB group (*p* < 0.001). This reduction in demand was mirrored by a substantial decrease in total opioid consumption; the mean intravenous morphine milligram equivalents administered in the ICNB group was 12.4 mg, representing a 38% reduction relative to the 20.1 mg required by patients in the standard care arm (*p* < 0.001) (Table 2).

Figure 2 illustrates four key findings from the mixed-effects model. First, all three groups followed the expected monotonic decline in pain over the first 24 postoperative hours, consistent with the significant main effect of time and indicating that the natural recovery trajectory was preserved across all analgesic strategies. Second, the three trajectories were clearly separated at every assessment point, with the ICNB curve consistently positioned below the local infiltration curve, which in turn was positioned below the opioid-only curve—an ordering confirmed by pairwise comparisons from the model’s estimated marginal means, consistent with the significant main effect of group. Third, the magnitude of the between-group differences was not constant across time but was largest during the early postoperative window (emergence and 6 h) and narrowed by 24 h—the statistical signature of the significant group × time interaction. Fourth, and most importantly, the ICNB group remained below the moderate-to-severe pain threshold (NRS = 4) at every assessment point including emergence, whereas the opioid-only group did not cross this threshold until between 12 and 24 h postoperatively. This visual contrast highlights the clinical importance of the observed differences: ICNB patients experienced bearable pain throughout recovery, whereas opioid-only patients spent the first half of the first postoperative day in the moderate-to-severe pain range (Figure 2).

### 3.3. Anatomical Characterization of Postoperative Pain

A detailed analysis of pain localization revealed distinct patterns that were largely unaffected by the analgesic intervention in terms of frequency, but were associated with differences in severity. Discomfort localized to the surgical port sites was nearly ubiquitous, reported by over 92% of patients in each group. However, the intensity of this incisional pain was attenuated in patients receiving the intercostal block, with a mean NRS of 3.5, compared to 4.2 for local infiltration and 4.8 for the control group (*p* < 0.001). More striking was the effect on posterior thoracic and interscapular pain, a common and often challenging component of the post-sympathectomy experience. Among the subset of patients reporting this specific pain, the mean intensity score was 5.9 in the opioid-only group, dropping to 5.1 with local infiltration and further down to 4.2 with ICNB (*p* < 0.001). This finding suggests that the intercostal block provides a field block effect that reaches deeper than the superficial port sites, mitigating the visceral or referred pain that contributes to patient discomfort (Table 3).

### 3.4. Safety, Tolerability, and Clinical Efficiency

The addition of regional anesthetic techniques did not introduce any new safety signals in this cohort. The overall rate of adverse events was low and comparable across the three groups of the study (*p* = 0.691). No instances of pneumothorax attributable to the intercostal block or signs of local anesthetic systemic toxicity were observed. There was a noticeable, though non-significant, trend toward a lower incidence of postoperative nausea and vomiting in the ICNB group (8.0% vs. 16.0% and 14.0%), which is what we would expect given the lower opioid doses these patients received. The incidence of compensatory hyperhidrosis, a recognized sequelae of sympathectomy, was stable at approximately 20% across all groups (*p* = 0.782) at 1-month follow-up; this short follow-up window may underestimate the ultimate CH incidence, which typically continues to manifest over 6–12 months postoperatively. From a health systems perspective, the improved analgesia translated into a modest but statistically meaningful reduction in the length of post-anesthesia care unit stay; patients receiving ICNB were discharged an average of one hour earlier than those in the control cohort (7.1 h vs. 8.2 h, *p* = 0.001) (Table 4).

### 3.5. Patient-Reported Outcomes and Quality of Recovery

The surgery itself led to large improvements in disease-specific QoL, irrespective of the perioperative pain management strategy. The mean QoL score on a 0–3 scale rose from a baseline of roughly 1.4 (indicating poor QoL) to between 2.7 and 2.8 postoperatively, a change that was both statistically significant (Group 1, *p* < 0.001; Group 2, *p* < 0.001; Group 3, *p* < 0.001) and clinically meaningful. Over 85% of patients in the ICNB group achieved a reduction in two or more grades on the HDSS (all *p* < 0.001 for HDSS change from baseline), a benchmark that confirms the effectiveness of the surgical intervention itself. However, the patient experience of recovery was clearly enhanced by better pain control. The median time to resumption of normal daily activities was just 3 days for patients in the ICNB group, compared to 4 days for the local infiltration group and 5 days for the opioid-only group (*p* = 0.008). This acceleration of functional recovery was reflected in satisfaction metrics; 91% of patients who received an intercostal block characterized themselves as “very satisfied” with the overall experience, a significantly higher endorsement rate than the 78% observed in the standard care arm (*p* = 0.044) (Table 5).

### 3.6. Independent Association with Pain Severity

**Figure 3:**
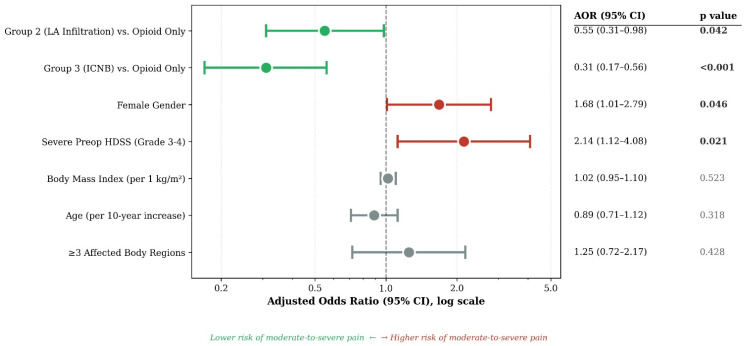
Forest plot of the multivariable logistic regression model for predictors of moderate-to-severe postoperative pain (NRS ≥ 4). All markers are adjusted odds ratios (AORs) with 95% confidence intervals from a single model, plotted on a logarithmic scale; the reference line at AOR = 1.0 denotes no association. For the analgesic-modality variable the reference category is Group 1 (opioid only), so the local-infiltration and ICNB markers represent the adjusted odds of moderate-to-severe pain for those groups relative to opioid-only analgesia—interpreted identically to the patient-level predictors, not as unadjusted between-group differences. Marker colour encodes the direction and statistical significance of each AOR: green, significantly reduced odds; red, significantly increased odds; grey, non-significant. The AOR (95% CI) and *p*-value are listed beside each row. The model showed acceptable calibration (Hosmer–Lemeshow *p* = 0.642) and discrimination (AUC = 0.742). LA, local anesthetic; ICNB, intercostal nerve block; HDSS, Hyperhidrosis Disease Severity Scale; CI, confidence interval.

A multivariable logistic regression model was constructed to determine which factors were independently associated with the likelihood of experiencing moderate-to-severe postoperative pain (NRS ≥ 4). The model was adjusted for age, gender, BMI, disease severity, and the specific analgesic intervention. After controlling for these variables, the intercostal nerve block emerged as the single strongest factor associated with reduced significant postoperative pain. Compared to patients receiving only systemic opioids, those in the ICNB group had 69% lower adjusted odds of reporting moderate-to-severe pain (AOR 0.31, 95% CI 0.17–0.56; *p* < 0.001). The local infiltration technique also offered a protective benefit, nearly halving the odds of significant pain (AOR 0.55, 95% CI 0.31–0.98; *p* = 0.042). In addition to the choice of analgesia, female gender (AOR 1.68, *p* = 0.046) and a high preoperative HDSS grade indicating severe disease (AOR 2.14, *p* = 0.021) were independently associated with an increased risk of experiencing a more painful postoperative course (Table 6; Figure 3 presents the same results as a forest plot).

Figure 3 presents the adjusted odds ratios (AORs) from the single multivariable logistic regression model as a forest plot. Every marker in the plot—including the two analgesic-group indicators—is an adjusted odds ratio estimated simultaneously within one model, expressing that variable’s independent effect on the odds of moderate-to-severe pain. The group rows are therefore the odds of moderate-to-severe pain for patients assigned to local infiltration or to ICNB relative to the opioid-only reference category, holding all other covariates constant; they are read on exactly the same scale and in exactly the same way as the patient-level predictors, and they are not unadjusted between-group comparisons of mean pain scores. Both regional strategies lie to the left of the no-effect line (AOR = 1): ICNB (AOR 0.31, 95% CI 0.17–0.56) is the strongest and most precisely estimated association, whereas local infiltration (AOR 0.55, 95% CI 0.31–0.98) only marginally excludes the null. Because the confidence intervals for the two regional techniques overlap, the model does not establish that ICNB is statistically superior to local infiltration; it indicates only that each is independently associated with lower odds of moderate-to-severe pain than opioid-only analgesia, with the ICNB estimate carrying the least uncertainty. Female gender (AOR 1.68, 95% CI 1.01–2.79) and severe preoperative HDSS (AOR 2.14, 95% CI 1.12–4.08) fall to the right of the reference line but with wide intervals whose lower bounds barely exclude 1; given this imprecision and the multiple-comparison correction, they are best regarded as hypothesis-generating rather than confirmed predictors. The remaining covariates (age, BMI, number of affected body regions) straddle AOR = 1, indicating no independent association after adjustment.

### 3.7. Sensitivity Analysis by Enrollment Year

To address concerns about temporal confounding from the gradual institutional adoption of ICNB during the enrollment period, we performed a sensitivity analysis stratified by year of enrollment (2018, 2019, and 2020). The distribution of patients by year and group is summarized below, with the proportion of ICNB cases increasing modestly over time as familiarity with the technique grew. The primary outcome—peak NRS—was re-analyzed using a linear mixed-effects model that additionally included enrollment year as a categorical fixed effect; the proportion of patients with moderate-to-severe pain was re-examined within each year stratum. The direction and approximate magnitude of the between-group differences were preserved across all three years (Table 7), supporting the robustness of the primary findings to temporal confounding.

## 4. Discussion

Our main finding is that the addition of a thoracoscopic-guided intercostal nerve block to a standardized systemic opioid regimen improved postoperative pain control, reduced opioid use, and shortened recovery time in patients undergoing BTS for primary focal hyperhidrosis. To our knowledge, this is the first controlled comparison of three different analgesic protocols in this specific ambulatory thoracic surgery setting.

Three figures support these findings. The participant flow diagram (Figure 1) documents that the analytic cohort represents 96.2% of all eligible patients screened, with no loss to follow-up across any of the three groups; this completeness reduces the risk that selective attrition contributed to the observed between-group differences, although it does not address the underlying allocation bias inherent to the non-randomized design. The pain trajectory plot (Figure 2) shows that the analgesic advantage of ICNB was not confined to a single time point but persisted across the entire 24 h observation window, with the ICNB curve remaining below the moderate-to-severe pain threshold (NRS ≥ 4) at every assessment—including the immediate post-emergence period when pain is typically most severe and rescue analgesia is most likely to be administered. Because both regional techniques were administered intraoperatively, before emergence from anesthesia, their analgesic effect was already established at the first postoperative assessment; the separation of the pain trajectories at emergence therefore reflects the pre-emptive timing of the interventions rather than any pre-existing between-group difference, consistent with the balanced baseline characteristics shown in Table 1. This is consistent with the pharmacokinetics of bupivacaine 0.25%, which has a clinical duration of action of approximately 6–8 h when used for peripheral nerve blockade, and aligns with the linear mixed-effects model’s significant group × time interaction, indicating that the magnitude of the benefit varied across the postoperative course rather than reflecting a uniform shift. Finally, the forest plot (Figure 3) shows that the ICNB association with reduced moderate-to-severe pain (AOR 0.31) is not only the strongest in the model but also the most precisely estimated, with the narrowest confidence interval among the protective associations; this precision strengthens confidence in the point estimate even after adjustment for demographic and clinical covariates.

Postoperative pain following BTS, while generally self-limiting, remains a near-universal experience that has received surprisingly little dedicated investigation. In the large series by Andrade Filho and colleagues, which included 1731 patients, postoperative pain was documented in 97.4% of cases, a figure that aligns closely with the 98–99% incidence observed across all groups in the present study [14]. Similarly, de Campos and coworkers reported that 90% of patients experienced some degree of thoracic pain following video-assisted thoracoscopic sympathectomy, with severe pain (NRS 8–10) occurring in 9.8% of cases [38]. Moya and colleagues noted pain during the first postoperative month in 80.7% of their 458 patients, with 7% reporting discomfort persisting beyond two months [39]. The fact that only a quarter of our control group required rescue analgesia despite nearly universal pain reports suggests that for many patients the discomfort, while present, is tolerable. However, a substantial minority—nearly three-quarters of the opioid-only group—experienced pain that reached the moderate-to-severe threshold (NRS ≥ 4), indicating a clear unmet need for more effective analgesia.

The apparent superiority of ICNB over both systemic opioids alone and simple port-site infiltration was evident across all measured domains. Patients receiving ICNB reported peak pain scores that were, on average, 1.4 points lower than controls and 0.8 points lower than the local infiltration group on the 11-point NRS. The 1.4-point reduction relative to opioid-only analgesia approaches or meets accepted thresholds for a minimal clinically important difference (MCID) in acute postoperative pain, which has been estimated at approximately 1 point (10 mm on a 100 mm visual analog scale) by Myles and colleagues [40] and shown to vary with baseline pain intensity in a systematic review by Olsen and colleagues [41]; it should be noted, however, that no absolute point-change MCID has been validated specifically for thoracic surgery, where a relative 30% improvement has been proposed as the threshold for meaningful symptom recovery after video-assisted thoracoscopic surgery [42]. The 0.8-point advantage over local infiltration, by contrast, is of more uncertain clinical significance. These findings are consistent with the work of Taylor and colleagues, who demonstrated that intercostal blockade provides effective analgesia following video-assisted thoracoscopic procedures, and extend those observations specifically to the BTS population [31]. More recently, a randomized trial by Elhouty and colleagues comparing rhomboid intercostal block to serratus anterior plane block for thoracoscopic sympathectomy reported similarly favorable analgesic outcomes, with both regional techniques significantly reducing pain scores and opioid requirements relative to controls [34]. In that study, the RIB group achieved a mean VAS of 0 at 1 h postoperatively compared to 0.23 in the SAPB group and 1.35 in controls, with time to first rescue analgesia prolonged to 730 min versus 40 min in the control group (*p* < 0.001) [34]. Their finding that intercostal-directed blocks offer particular advantage aligns with our observation that ICNB was associated with greater pain reduction than local infiltration, which provides only superficial anesthesia at the skin incision sites. Furthermore, a recent trial by Minqiang and colleagues evaluating opioid-reduced anesthesia with intercostal nerve block for thoracoscopic sympathectomy found that while the technique was safe and effective, it did not demonstrate clinical advantage over standard opioid-based anesthesia in terms of pain scores or adverse events [32]. This contrasts with our findings and may reflect differences in the specific regional technique employed, the timing of block administration, or the patient population studied. Furthermore, their use of a laryngeal mask airway and spontaneous ventilation [32] may have altered the pain experience compared to our intubated, mechanically ventilated cohort.

There is a clear anatomical and physiological reason for the observed advantage of ICNB. Local anesthetic infiltration at the port sites, while simple and safe, anesthetizes only the superficial layers of the chest wall at the point of trocar entry. As noted in a comprehensive review by Stamenkovic and colleagues, wound infiltration is most effective when applied to all tissue layers involved in the surgical incision, and superficial infiltration alone may be insufficient for procedures generating deeper somatic pain [28]. The ICNB, by depositing local anesthetic directly into the intercostal space adjacent to the intercostal neurovascular bundle near the paravertebral gutter, provides a field block that encompasses the intercostal nerves carrying afferent signals from the operative field and the deeper musculoskeletal structures of the chest wall. In our analysis of pain localization, posterior thoracic pain—reported by over 70% of patients—was the most intense component of the postoperative pain experience, with mean NRS scores reaching 5.9 in the control group. The fact that ICNB was associated with reduced posterior thoracic pain intensity to a mean of 4.2 provides supportive evidence that this technique addresses the dominant source of post-sympathectomy discomfort.

Our observation that ICNB was associated with faster functional recovery: patients returned to normal activities about two days earlier than controls, which is clinically important for fast-track and ambulatory thoracic surgery. As Molins and colleagues demonstrated, adequate postoperative analgesia is a cornerstone of successful outpatient thoracic surgical programmes, with their series of 300 patients showing that effective pain control enabled same-day discharge in the majority of cases [29]. The reduced length of stay in the ICNB group—approximately one hour shorter than controls—supports the link between better pain control and shorter hospital stay. This is also in line with the broader enhanced recovery after surgery (ERAS) literature, which emphasizes the importance of multimodal, opioid-sparing analgesic strategies in facilitating early mobilization and discharge [28,43]. The ERAS Society and European Society of Thoracic Surgeons guidelines for lung surgery explicitly recommend regional analgesia and opioid minimization as core components of enhanced recovery [43], and the procedure-specific PROSPECT recommendations for video-assisted thoracoscopic surgery likewise prioritize regional techniques with opioids reserved for rescue [44]. Within such pathways, a simple, low-cost, thoracoscopically delivered block such as ICNB could be readily integrated into ambulatory and short-stay thoracic surgery programmes, where avoidance of opioid-related side effects is particularly advantageous for same-day discharge [25,37].

ICNB delivered better analgesia without a measurable safety trade-off. We observed no technique-specific complications such as pneumothorax requiring intervention or signs of local anesthetic systemic toxicity. This safety profile is consistent with the experience of Han and colleagues, who performed thoracoscopic sympathetic nerve block in 302 patients and reported minor pneumothorax in 2.0% of cases, none requiring chest tube insertion, and temporary ptosis in 3.4% [45]. Yoo and coworkers similarly documented only two cases of minor pneumothorax (3.85%) among 52 patients undergoing thoracoscopic sympathetic nerve block, both resolving spontaneously [19]. The overall incidence of adverse events in our study was comparable across the three groups, with a non-significant trend toward less postoperative nausea and vomiting in the ICNB cohort—a finding that almost certainly reflects the substantially lower doses of opioids these patients received.

The incidence of compensatory hyperhidrosis was stable at approximately 20% across all groups at 1-month follow-up, confirming that the choice of perioperative analgesic technique does not influence this sympathetically mediated phenomenon. This rate is lower than typically reported in the long-term literature (50–90%) [14,16,19] and almost certainly reflects the brief follow-up window of our study; CH manifestation typically continues over 6–12 months postoperatively. The 1-month rate reported here should therefore be interpreted as provisional. Among contemporary series with comparable surgical technique (limited, caudally targeted sympathicotomies at the R3 or R4 level), Romano et al. reported a CH rate of 25% [46], in line with our findings. Our finding that analgesic modality does not influence CH provides reassurance for clinicians considering adoption of ICNB.

The large improvement in disease-specific QoL across all three groups—with mean QoL scores rising from approximately 1.4 preoperatively to between 2.7 and 2.8 postoperatively—is consistent with the well-established benefits of BTS documented in earlier large series [7,16,47]. The fact that our QoL outcomes were comparable across analgesic groups confirms that the surgical procedure itself, rather than the perioperative pain management strategy, is the primary driver of long-term patient satisfaction.

The multivariable logistic regression model provided additional insight into the factors independently associated with moderate-to-severe postoperative pain. After adjusting for relevant covariates, ICNB remained the strongest protective factor, associated with a 69% reduction in the adjusted odds of experiencing NRS ≥ 4. As displayed in the forest plot (Figure 3), the visual hierarchy of effect sizes places ICNB first (AOR 0.31), followed by local infiltration (AOR 0.55), with both regional techniques showing confidence intervals that do not cross the null reference line, supporting the conclusion that the analgesic strategy itself—rather than patient-level characteristics—is the dominant modifiable determinant of postoperative pain in this surgical context. Notably, female gender (AOR 1.68, *p* = 0.046) and severe preoperative HDSS scores (AOR 2.14, *p* = 0.021) were also independently associated with increased pain risk. These two findings are borderline significant—as evident in Figure 3 from confidence intervals whose lower bounds (1.01 and 1.12, respectively) only just exclude the reference line—and may not survive correction for multiple comparisons; they should therefore be regarded as hypothesis-generating. The association with female gender is well-documented across numerous surgical populations and likely reflects a complex interplay of biological, psychological, and sociocultural factors [28]. The finding that patients with more severe baseline hyperhidrosis experience greater postoperative pain is intriguing and may suggest a generalized upregulation of sympathetic or nociceptive pathways in this subgroup [48], though this remains speculative.

Several limitations of this study merit careful and substantive consideration. First, and most importantly, the observational, non-randomized design introduces real potential for allocation bias and unmeasured confounding. Group assignment was determined by the attending anesthesiologist based on clinical and practical considerations rather than by randomization, and although demographic and clinical baseline characteristics were balanced across the three cohorts, residual confounding by factors such as anesthesiologist experience, intraoperative dynamics, and temporal practice trends cannot be excluded. The proportion of patients receiving ICNB increased over the enrollment period, and although a sensitivity analysis stratified by enrollment year did not materially alter the conclusions, the possibility that secular improvements in perioperative care contributed to the observed benefit cannot be fully ruled out. A randomized controlled trial would provide more definitive evidence and should be a priority for future investigation. We also did not measure or adjust for psychosocial and behavioral modifiers of pain perception—such as preoperative anxiety, pain catastrophizing, and individual differences in baseline pain sensitivity—any of which could have influenced postoperative pain reporting independently of the analgesic technique. Second, complete blinding of outcome assessors was not feasible because the operative record documented the regional technique used; although we used standardized assessment forms that did not display the analgesic protocol, observer bias cannot be entirely excluded. Relatedly, patient-reported satisfaction may be vulnerable to expectation bias: patients who were aware of receiving a more elaborate regional technique may have perceived their care as more advanced, potentially inflating satisfaction independently of any true analgesic benefit. In addition, performance bias cannot be excluded, since anesthesiologists and perioperative teams more experienced with ICNB may have differed systematically in other, unmeasured aspects of perioperative management. Third, and importantly, none of the three groups received scheduled baseline multimodal non-opioid analgesia (paracetamol, NSAIDs); this reflects institutional practice during the study period but is fundamentally at odds with current ERAS recommendations. Consequently, the opioid-only control arm does not represent a contemporary standard-of-care comparator, and the magnitude of the apparent benefit of ICNB is likely overestimated relative to what would be observed against a modern multimodal regimen. In current practice, ICNB would rarely be compared against an opioid-only protocol without scheduled non-opioid analgesics, and this limitation materially constrains the external validity of our findings. The observed effect sizes should therefore be interpreted with this asymmetry firmly in mind. Fourth, the study was conducted at a single high-volume thoracic surgery center; the generalizability of our findings to lower-volume settings remains uncertain. Fifth, our follow-up period was limited to the immediate perioperative window and the first postoperative month, precluding assessment of potential influence on chronic post-sympathectomy pain and almost certainly underestimating the ultimate incidence of compensatory hyperhidrosis. Sixth, although we applied a Benjamini–Hochberg false discovery rate correction to secondary endpoints, the large number of statistical comparisons performed means that some borderline-significant findings (notably the female-gender and HDSS associations in the multivariable model) should be regarded as hypothesis-generating rather than confirmatory.

## 5. Conclusions

In conclusion, our prospective observational study indicates that adding a thoracoscopic-guided intercostal nerve block to systemic opioid analgesia improves postoperative pain control, reduces opioid use, and shortens functional recovery in patients undergoing bilateral thoracoscopic sympathicotomy for primary focal hyperhidrosis. The technique is safe, technically straightforward, and can be readily incorporated into the surgical workflow. Based on these findings, ICNB may be considered a promising perioperative analgesic option for patients undergoing BTS, particularly in ambulatory or short-stay thoracic surgery programmes, although its role relative to contemporary multimodal regimens remains to be established. However, confirmation of these findings in a randomized controlled trial—ideally with multimodal non-opioid analgesia in the control arm—is warranted before ICNB can be definitively recommended as standard practice.

## Figures and Tables

**Table 1:** Baseline Demographic and Clinical Characteristics of Study Participants.

Characteristic	Group 1: Opioid Only (*n* = 100)	Group 2: LA Infiltration (*n* = 100)	Group 3: ICNB (*n* = 100)	*p* Value
Age (years), mean ± SD	32.4 ± 8.7	33.1 ± 9.2	31.8 ± 8.9	0.612
Body Mass Index (kg/m^2^), mean ± SD	24.8 ± 3.4	24.3 ± 3.1	24.1 ± 3.3	0.319
Gender, *n* (%)				
Male	36 (36.0%)	38 (38.0%)	42 (42.0%)	0.684
Female	64 (64.0%)	62 (62.0%)	58 (58.0%)	
Number of Affected Body Regions, *n* (%)				
1–2 Regions	52 (52.0%)	48 (48.0%)	54 (54.0%)	0.703
≥3 Regions	48 (48.0%)	52 (52.0%)	46 (46.0%)	
Affected Anatomical Sites, *n* (%)				
Palmar	68 (68.0%)	71 (71.0%)	65 (65.0%)	0.672
Axillary	82 (82.0%)	79 (79.0%)	76 (76.0%)	0.582
Facial	12 (12.0%)	15 (15.0%)	18 (18.0%)	0.499
Plantar	54 (54.0%)	58 (58.0%)	52 (52.0%)	0.702
Pre-op HDSS Grade 3–4 (Severe), *n* (%)	82 (82.0%)	79 (79.0%)	84 (84.0%)	0.651
Prior Conservative Therapy, *n* (%)	48 (48.0%)	51 (51.0%)	53 (53.0%)	0.772
Positive Family History, *n* (%)	34 (34.0%)	31 (31.0%)	33 (33.0%)	0.891
Pre-op QoL Score (0–3), mean ± SD	1.42 ± 0.68	1.38 ± 0.71	1.45 ± 0.65	0.758

Continuous variables are expressed as mean ± standard deviation (SD) and were analyzed using One-Way Analysis of Variance (ANOVA) with post hoc test (Tukey Honestly Significant Difference test, HSD). Categorical variables are presented as absolute numbers with percentages and were compared using the Chi-square test. Preoperative Quality of Life (QoL) was assessed on a scale where 0 indicates extremely poor and 3 indicates excellent: LA, Local Anesthetic; ICNB, Intercostal Nerve Block; HDSS, Hyperhidrosis Disease Severity Scale.

**Table 2:** Postoperative Pain Trajectory, Intensity, and Analgesic Requirements.

Variable	Group 1: Opioid Only (*n* = 100)	Group 2: LA Infiltration (*n* = 100)	Group 3: ICNB (*n* = 100)	*p* (F; η^2^, 95% CI) or *p* (χ^2^(2); Cramér’s V)
Peak Pain Intensity (NRS 0–10), mean ± SD	5.2 ± 2.1	4.6 ± 1.9	3.8 ± 1.7	<0.001 (F(2, 297) = 24.6; η^2^ = 0.14, 95% CI 0.08–0.21)
NRS at 6 h Post-op, mean ± SD	4.8 ± 2.0	4.1 ± 1.8	3.2 ± 1.5	<0.001 (F(2, 297) = 19.8; η^2^ = 0.12, 95% CI 0.06–0.19)
NRS at 12 h Post-op, mean ± SD	3.9 ± 1.8	3.3 ± 1.6	2.5 ± 1.4	<0.001 (F(2, 297) = 17.4; η^2^ = 0.11, 95% CI 0.05–0.18)
NRS at 24 h Post-op, mean ± SD	3.1 ± 1.6	2.6 ± 1.5	2.0 ± 1.2	<0.001 (F(2, 297) = 14.1; η^2^ = 0.09, 95% CI 0.04–0.16)
Moderate-to-Severe Pain (NRS ≥4), *n* (%)	74 (74.0%)	61 (61.0%)	42 (42.0%)	<0.001 (χ^2^(2) = 21.3; Cramér’s V = 0.27)
Pain Duration, *n* (%)				
Less than 1 week	22 (22.2%)	31 (31.6%)	38 (38.8%)	0.023 †
1 to 2 weeks	31 (31.3%)	34 (34.7%)	36 (36.7%)	
2 weeks to 1 month	28 (28.3%)	22 (22.4%)	18 (18.4%)	
More than 1 month	18 (18.2%)	11 (11.2%)	6 (6.1%)	
Trend test (Cochran–Armitage)				0.004 ‡
Rescue Analgesia Required, *n* (%)	68 (68.0%)	57 (57.0%)	42 (42.0%)	<0.001 (χ^2^(2) = 14.8; Cramér’s V = 0.22)
Total Opioid Consumption (MME, mg), mean ± SD	20.1 ± 6.3	17.5 ± 5.9	12.4 ± 4.8	<0.001 (F(2, 297) = 31.2; η^2^ = 0.17, 95% CI 0.10–0.24)

Data are presented as mean ± standard deviation (SD) or absolute numbers with valid percentages. The longitudinal NRS trajectory across the four assessment time points was analyzed using a linear mixed-effects model with group, time, and group × time interaction as fixed effects and patient as a random intercept (see Section 2.6); pairwise between-group comparisons at each time point were obtained from estimated marginal means with Tukey adjustment. Cross-sectional outcomes (peak pain, total opioid consumption) were compared using One-Way ANOVA with post hoc Tukey HSD. Categorical variables were compared using the Chi-square test. † Chi-square test for the overall 4 × 3 contingency. ‡ Cochran–Armitage linear-by-linear trend test for monotonic association between ordered pain duration category and analgesic group, weighting categories 1–4. Pain duration percentages are based on patients reporting any postoperative pain (*n* = 99, 98, 98 in Groups 1, 2, 3 respectively). ANOVA, Analysis of Variance; F(df1, df2), F statistic with numerator and denominator degrees of freedom; eta-squared, effect size for ANOVA; chi-square, Chi-square statistic; Cramer’s V, effect size for the Chi-square test; CI, confidence interval; SD, standard deviation. Continuous outcomes were compared by One-Way ANOVA (effect size: eta-squared) and categorical outcomes by the Chi-square test (effect size: Cramer’s V); the longitudinal NRS trajectory was analyzed by a linear mixed-effects model, NRS, Numerical Rating Scale; MME, Morphine Milligram Equivalents.

**Table 3:** Localization and Intensity of Postoperative Pain by Study Group.

Pain Localization	Group 1: Opioid Only	Group 2: LA Infiltration	Group 3: ICNB	*p* (F; η^2^, 95% CI) or *p* (χ^2^(2); Cramér’s V)
Any Pain Location, *n* (%)				
Surgical Port Sites (Incision)	94 (94.9%)	92 (93.9%)	91 (92.9%)	0.853 (χ^2^(2) = 0.32; Cramér’s V = 0.03)
Anterior Chest Wall (Retrosternal)	42 (42.4%)	40 (40.8%)	38 (38.8%)	0.872 (χ^2^(2) = 0.27; Cramér’s V = 0.03)
Posterior Thorax (Interscapular/Spinal)	78 (78.8%)	73 (74.5%)	70 (71.4%)	0.547 (χ^2^(2) = 1.21; Cramér’s V = 0.06)
Mean NRS by Location, mean ± SD				
Surgical Port Sites	4.8 ± 1.9 (*n* = 94)	4.2 ± 1.7 (*n* = 92)	3.5 ± 1.5 (*n* = 91)	<0.001 (F(2, 274) = 13.4; η^2^ = 0.089, 95% CI 0.032–0.154)
Anterior Chest Wall	4.5 ± 2.1 (*n* = 42)	4.1 ± 1.8 (*n* = 40)	3.3 ± 1.6 (*n* = 38)	0.004 (F(2, 117) = 4.3; η^2^ = 0.068, 95% CI 0.002–0.160)
Posterior Thorax	5.9 ± 2.2 (*n* = 78)	5.1 ± 2.0 (*n* = 73)	4.2 ± 1.8 (*n* = 70)	<0.001 (F(2, 218) = 13.1; η^2^ = 0.108, 95% CI 0.038–0.184)

Frequencies are presented as the number of symptomatic patients reporting pain at a given site, with percentages calculated based on the total number of patients who experienced any postoperative pain in each group (Group 1: *n* = 99; Group 2: *n* = 98; Group 3: *n* = 98). Intensity scores are presented as mean Numerical Rating Scale (NRS) score ± standard deviation (SD) for those specific patients. Statistical comparisons for NRS scores utilized One-Way ANOVA with post hoc test (Tukey Honestly Significant Difference test, HSD). Significant post hoc differences (*p* < 0.05) were noted for Surgical Port Sites (G3 < G2 and G1) and Posterior Thorax (G3 < G2 and G1). Frequency comparisons across study groups used the Chi-square test (χ^2^(2)) with Cramér’s V as the effect size; NRS-by-location comparisons used One-Way ANOVA, reported as the F statistic with its degrees of freedom, F(df1, df2), with eta-squared (η^2^) and 95% CI as the effect size, and pairwise Cohen’s d (Group 3 vs. Group 1; Group 3 vs. Group 2) of 0.76 and 0.44 for surgical port sites, 0.64 and 0.47 for anterior chest wall, and 0.84 and 0.47 for posterior thorax. χ^2^(2), Chi-square statistic with 2 degrees of freedom; Cramér’s V, effect size for the Chi-square test; F(df1, df2), F statistic with numerator and denominator degrees of freedom; η^2^, eta-squared; CI, confidence interval, LA, Local Anesthetic; ICNB, Intercostal Nerve Block.

**Table 4:** Perioperative Complications and Clinical Efficiency Metrics.

Variable	Group 1: Opioid Only (*n* = 100)	Group 2: LA Infiltration (*n* = 100)	Group 3: ICNB (*n* = 100)	*p* (F; η^2^, 95% CI) or *p* (χ^2^(2); Cramér’s V)
Any Perioperative Complication, *n* (%)	42 (42.0%)	45 (45.0%)	39 (39.0%)	0.691 (χ^2^(2) = 0.74; Cramér’s V = 0.05)
Postoperative Nausea/Vomiting, *n* (%)	16 (16.0%)	14 (14.0%)	8 (8.0%)	0.194 (χ^2^(2) = 3.28; Cramér’s V = 0.10)
Transient Paresthesia/Numbness, *n* (%)	11 (11.0%)	13 (13.0%)	15 (15.0%)	0.705 (χ^2^(2) = 0.70; Cramér’s V = 0.05)
Compensatory Hyperhidrosis, *n* (%)	21 (21.0%)	23 (23.0%)	19 (19.0%)	0.782 (χ^2^(2) = 0.49; Cramér’s V = 0.04)
Local Anesthetic-Related Complication, *n* (%)	N/A	0 (0.0%)	0 (0.0%)	—
Length of Hospital Stay (hours), mean ± SD	8.2 ± 2.4	7.8 ± 2.1	7.1 ± 1.9	0.001 (F(2, 297) = 6.75; η^2^ = 0.043, 95% CI 0.007–0.093)

Data are presented as absolute numbers with percentages or mean ± standard deviation. Categorical variables were compared using the Chi-square test. Length of stay was compared using One-Way ANOVA with post hoc (Tukey Honestly Significant Difference test, HSD). Effect sizes are reported as Cramér’s V for categorical comparisons (Chi-square test, χ^2^(2)) and as eta-squared (η^2^) with 95% CI for the One-Way ANOVA, with the F statistic reported together with its degrees of freedom; for length of stay, pairwise Cohen’s d was 0.51 (Group 3 vs. Group 1) and 0.35 (Group 3 vs. Group 2). χ^2^(2), Chi-square statistic; Cramér’s V, effect size for the Chi-square test; F(df1, df2), F statistic with degrees of freedom; η^2^, eta-squared; CI, confidence interval, LA, Local Anesthetic; ICNB, Intercostal Nerve Block; N/A, Not Applicable.

**Table 5:** Quality of Life and Functional Recovery Outcomes.

Outcome Measure	Group 1: Opioid Only (*n* = 100)	Group 2: LA Infiltration (*n* = 100)	Group 3: ICNB (*n* = 100)	*p* (F; η^2^, 95% CI ) or *p* (χ^2^(2); Cramér’s V)
Post-op QoL Score (0–3), mean ± SD	2.68 ± 0.52	2.74 ± 0.48	2.82 ± 0.41	0.111 (F(2, 297) = 2.21; η^2^ = 0.015, 95% CI 0.000–0.048)
Post-op QoL “Excellent” (Score 3), *n* (%)	72 (72.0%)	78 (78.0%)	84 (84.0%)	0.124 (χ^2^(2) = 4.17; Cramér’s V = 0.12)
HDSS Improvement ≥2 Grades, *n* (%)	79 (79.0%)	82 (82.0%)	86 (86.0%)	0.428 (χ^2^(2) = 1.70; Cramér’s V = 0.08)
Return to Normal Activity (days), median (IQR)	5 (3–7)	4 (3–6)	3 (2–5)	0.008 (H(2) = 9.66; ε^2^ = 0.026)
Very Satisfied with Procedure, *n* (%)	78 (78.0%)	85 (85.0%)	91 (91.0%)	0.044 (χ^2^(2) = 6.25; Cramér’s V = 0.14)
Would Recommend Procedure, *n* (%)	96 (96.0%)	97 (97.0%)	99 (99.0%)	0.571 (χ^2^(2) = 1.12; Cramér’s V = 0.06)

Postoperative QoL score and HDSS improvement (between-group comparisons) were analyzed by One-Way ANOVA with post hoc Tukey HSD; categorical outcomes (QoL ≥ 3, satisfaction, recommendation) by Chi-square. Return to normal activity (skewed, reported as median with interquartile range) was compared between groups using the Kruskal–Wallis test. Within-group preoperative-to-postoperative changes in QoL and HDSS were analyzed by Wilcoxon signed-rank test on a per-patient basis. For continuous outcomes the F statistic with its degrees of freedom and eta-squared (η^2^) with 95% CI are reported; for the Kruskal–Wallis comparison the H statistic with its degrees of freedom, H(2), and epsilon-squared (ε^2^) are reported; categorical comparisons report the Chi-square statistic (χ^2^(2)) with Cramér’s V. F(df1, df2), F statistic with numerator and denominator degrees of freedom; η^2^, eta-squared; H(2), Kruskal–Wallis H statistic with 2 degrees of freedom; ε^2^, epsilon-squared (effect size for the Kruskal–Wallis test); χ^2^(2), Chi-square statistic; Cramér’s V, effect size for the Chi-square test; CI, confidence interval, HDSS, Hyperhidrosis Disease Severity Scale; QoL, Quality of Life.

**Table 6:** Multivariable Logistic Regression for Predictors of Moderate-to-Severe Pain (NRS ≥ 4).

Variable	Adjusted Odds Ratio (AOR)	95% Confidence Interval (95% CI)	*p* Value
Analgesic Modality			
Group 1 (Opioid Only)	1.00 (Reference)	—	—
Group 2 (LA Infiltration)	0.55	0.31–0.98	0.042
Group 3 (ICNB)	0.31	0.17–0.56	<0.001
Patient Characteristics			
Female Gender	1.68	1.01–2.79	0.046
Severe Pre-op HDSS (Grade 3–4)	2.14	1.12–4.08	0.021
Body Mass Index (per 1 kg/m^2^)	1.02	0.95–1.10	0.523
Age (per 10-year increase)	0.89	0.71–1.12	0.318
≥3 Affected Body Regions	1.25	0.72–2.17	0.428

Multivariable logistic regression analysis. The model demonstrated acceptable calibration (Hosmer–Lemeshow goodness-of-fit test, *p* = 0.642) and discrimination (Area Under the Receiver Operating Characteristic Curve, AUC = 0.742). AOR, Adjusted Odds Ratio; CI, Confidence Interval; LA, Local Anesthetic; ICNB, Intercostal Nerve Block; HDSS, Hyperhidrosis Disease Severity Scale.

**Table 7:** Sensitivity Analysis: Primary Outcomes Stratified by Enrollment Year.

Characteristic	Group 1: Opioid Only (*n* = 100)	Group 2: LA Infiltration (*n* = 100)	Group 3: ICNB (*n* = 100)	*p* (F; η^2^, 95% CI ) or *p* (χ^2^(2); Cramér’s V)
Patients enrolled, n				
2018	39	34	27	—
2019	34	33	33	—
2020	27	33	40	—
Peak NRS, mean ± SD				
2018	5.3 ± 2.0	4.7 ± 1.8	3.9 ± 1.6	0.012 (F(2, 97) = 4.66; η^2^ = 0.088, 95% CI 0.005–0.195)
2019	5.1 ± 2.2	4.6 ± 2.0	3.8 ± 1.7	0.029 (F(2, 97) = 3.67; η^2^ = 0.070, 95% CI 0.001–0.172)
2020	5.2 ± 2.1	4.5 ± 1.9	3.7 ± 1.7	0.007 (F(2, 97) = 5.25; η^2^ = 0.098, 95% CI 0.009–0.208)
Moderate-to-severe pain, *n* (%)				
2018	29 (74.4%)	21 (61.8%)	12 (44.4%)	0.027 (χ^2^(2) = 7.22; Cramér’s V = 0.27)
2019	25 (73.5%)	20 (60.6%)	14 (42.4%)	0.034 (χ^2^(2) = 6.76; Cramér’s V = 0.26)
2020	20 (74.1%)	20 (60.6%)	16 (40.0%)	0.022 (χ^2^(2) = 7.63; Cramér’s V = 0.28)
Mixed-effects model (year-adjusted)				
ICNB vs. Opioid (β coefficient)	Reference	—	−1.41	<0.001 (95% CI −1.86 to −0.96; t = −6.14)
Year (per-year, categorical)	—	—	—	0.872 (F(2, 294) = 0.14)

Sensitivity analysis stratified by enrollment year. Patient counts demonstrate the gradual increase in ICNB allocation over the study period (from 27% of patients in 2018 to 40% in 2020), reflecting institutional uptake of the technique. Peak NRS comparisons within each year stratum were performed by One-Way ANOVA; moderate-to-severe pain proportions by Chi-square. The year-adjusted mixed-effects model preserved the magnitude and direction of the ICNB protective effect (β = −1.41 NRS points, *p* < 0.001), with no significant main effect of enrollment year (*p* = 0.872), indicating that secular trends in perioperative care do not account for the observed between-group differences. Within-year Peak NRS comparisons are reported as the F statistic with its degrees of freedom and eta-squared (η^2^) with 95% CI; within-year moderate-to-severe pain proportions as the Chi-square statistic (χ^2^(2)) with Cramér’s V. For the year-adjusted mixed-effects model, the ICNB versus opioid-only contrast is reported as the β coefficient with its 95% CI and t statistic, and the main effect of enrollment year as an F test with its degrees of freedom. F(df1, df2), F statistic with degrees of freedom; η^2^, eta-squared; χ^2^(2), Chi-square statistic; Cramér’s V, effect size for the Chi-square test; CI, confidence interval, LA, Local Anesthetic; ICNB, Intercostal Nerve Block; NRS, Numerical Rating Scale.

## Data Availability

The data presented in this study are available on request from the corresponding author due to privacy and ethical restrictions.

## Supplementary Materials

The following supporting information can be downloaded at: https://www.mdpi.com/article/10.3390/jcm15145755/s1, Table S1: Distribution of Group Allocation by Enrollment Month; Table S2: Raw and Benjamini-Hochberg FDR-adjusted *p*-values for secondary outcomes. File S1: STROBE Statement—Checklist of Items That Should Be Included in Reports of Cohort Studies.
