# Supplementary material for: Intercostal Nerve Block for Bilateral Thoracoscopic Sympathectomy: A Prospective Observational Cohort Comparison of Three Analgesic Protocols
Source: J Clin Med. 2026 Jul 22;15(14):5755. doi: 10.3390/jcm15145755 (PMC13412612; doi:10.3390/jcm15145755)
Supplement: Supplementary file 1 [file jcm-15-05755-s001.zip › Supplementary File S1.pdf]

Supplementary File S1. STROBE Statement — Checklist of Items That Should Be Included in Reports of Cohort Studies

STROBE Version: This checklist follows the official STROBE Statement (Strengthening the Reporting of Observational Studies in Epidemiology), updated October 2007. Available at: *www.strobe-statement.org*.

Item No	Section/Topic	Recommendation	Reported on (page/section)
		Title and Abstract	
1(a)	**Title**	Indicate the study's design with a commonly used term in the title or the abstract.	Title formed to include 'Prospective Observational Cohort Comparison'; abstract states 'prospective observational cohort study'.
1(b)	**Abstract**	Provide in the abstract an informative and balanced summary of what was done and what was found.	Section 1 (Abstract): Background/Objectives, Methods, Results, Conclusions structure with all key findings.
		Introduction	
2	**Background/Rationale**	Explain the scientific background and rationale for the investigation being reported.	Section 1 (Introduction): epidemiology of PFH, BTS as treatment, postoperative pain burden, rationale for ICNB.
3	**Objectives**	State specific objectives, including any prespecified hypotheses.	Section 1, final paragraph: explicit objectives and hypothesis that ICNB is superior to local infiltration and opioid alone.
		Methods	
4	**Study Design**	Present key elements of study design early in the paper.	Section 2.1: prospective observational cohort, single-center, parallel three-group comparison, no randomization.
5	**Setting**	Describe the setting, locations, and relevant dates, including periods of recruitment, exposure, follow-up, and data collection.	Section 2.1: Institute for Pulmonary Diseases of Vojvodina, Sremska Kamenica, Serbia; February 2018 – December 2020 enrollment; 1-month follow-up.
6(a)	**Participants — Eligibility Criteria**	Cohort study—Give the eligibility criteria, and the sources and methods of selection of participants.	Section 2.2: detailed inclusion/exclusion criteria; consecutive eligible patients screened.
6(b)	**Participants — Methods of Follow-up**	Cohort study—For matched studies, give matching criteria and number of exposed and unexposed.	Not applicable — matched design not used. All n=300 participants completed 1-month follow-up (loss = 0%).
7	**Variables**	Clearly define all outcomes, exposures, predictors, potential confounders, and effect modifiers. Give diagnostic criteria, if applicable.	Section 2.5 (outcome definitions: NRS, MME, complications, QoL, satisfaction); Section 2.6 (covariates for multivariable model); Section 2.4 (HDSS classification).
8*	**Data Sources / Measurement**	For each variable of interest, give sources of data and details of methods of assessment (measurement). Describe comparability of assessment methods if there is more than one group.	Section 2.5: standardized data collection forms, NRS at four time points, MME calculated by CDC opioid conversion guide [33], 1.5-point MCID per Farrar et al. [33], identical assessment protocol across all three groups.
9	**Bias**	Describe any efforts to address potential sources of bias.	Section 2.6 (sensitivity analysis stratified by enrollment year addressing temporal confounding); Section 4.1 (limitations: explicit acknowledgment of allocation bias from non-randomization, lack of blinding, single-center design).
10	**Study Size**	Explain how the study size was arrived at.	Section 2.7: sample size calculation based on detecting a 1.5-point difference in NRS (MCID per Farrar et al. [39]) with α=0.05, power=0.80, anticipated SD=2.0; required n≈90 per group; recruited 100 per group for buffer.

Item No	Section/Topic	Recommendation	Reported on (page/section)
11	**Quantitative Variables**	Explain how quantitative variables were handled in the analyses. If applicable, describe which groupings were chosen and why.	Section 2.6: continuous variables tested with Shapiro-Wilk; means±SD if normal, median (IQR) if non-normal; HDSS treated as ordinal with severity dichotomization (Grade 1-2 vs 3-4) for multivariable model based on clinical convention.
12(a)	**Statistical Methods**	Describe all statistical methods, including those used to control for confounding.	Section 2.6: Linear mixed-effects model for longitudinal NRS with patient as random intercept; One-Way ANOVA for cross-sectional outcomes; Kruskal-Wallis for non-normal; Chi-square/Fisher's for categorical; Cochran-Armitage for ordinal categorical (pain duration); Wilcoxon signed-rank test for pre-post within-patient changes; multivariable logistic regression for moderate-to-severe pain.
12(b)	**Statistical Methods — Subgroups**	Describe any methods used to examine subgroups and interactions.	Section 2.6: pre-specified subgroup analyses by gender, baseline HDSS severity, and number of affected body regions; group × time interaction explicitly tested in mixed-effects model.
12(c)	**Statistical Methods — Missing Data**	Explain how missing data were addressed.	Section 2.6: 0% loss to follow-up; mixed-effects model handles within-subject missing time points using maximum likelihood (no imputation needed).
12(d)	**Statistical Methods — Loss to Follow-up**	Cohort study—If applicable, explain how loss to follow-up was addressed.	Section 3 (Results): all 300 enrolled patients completed 1-month follow-up; 0% loss to follow-up across all three groups.
12(e)	**Statistical Methods — Sensitivity Analyses**	Describe any sensitivity analyses.	Section 2.6 + Section 3.7 + Table 7: sensitivity analysis stratified by enrollment year (2018, 2019, 2020); year-adjusted linear mixed-effects model. Section 2.6: Benjamini-Hochberg FDR correction for 15 secondary outcome p-values (Supplementary Table S2).
		Results	
13(a)	**Participants — Numbers**	Report numbers of individuals at each stage of study—e.g., numbers potentially eligible, examined for eligibility, confirmed eligible, included in the study, completing follow-up, and analyzed.	Section 3.1 + Figure 1 (STROBE flow diagram): 312 screened, 12 excluded with reasons, 300 enrolled, 300 completed follow-up, 300 analyzed.
13(b)	**Participants — Reasons for Non-Participation**	Give reasons for non-participation at each stage.	Figure 1: 12 exclusions detailed (4 prior thoracic surgery, 3 BMI > 35, 2 chronic pain syndrome with regular analgesic use, 1 bupivacaine allergy, 2 declined participation).
13(c)	**Participants — Flow Diagram**	Consider use of a flow diagram.	Figure 1: STROBE flow diagram presented in Methods Section 2.2.
14(a)	**Descriptive Data — Characteristics**	Give characteristics of study participants (e.g., demographic, clinical, social) and information on exposures and potential confounders.	Section 3.1 + Table 1: age, sex, BMI, body region distribution, baseline HDSS severity, prior conservative therapy, family history, preoperative QoL — all presented per group with p-values for between-group comparison.
14(b)	**Descriptive Data — Missing Data**	Indicate number of participants with missing data for each variable of interest.	Table 1 footnote and Section 3.1: complete data for all 300 participants on all baseline variables; 0% missing.
14(c)	**Descriptive Data — Follow-up Time**	Cohort study—Summarize follow-up time (e.g., average and total amount).	Section 3 (Results): all participants followed for 1 month postoperatively (24-h perioperative monitoring + 1-month outpatient assessment).

Item No	Section/Topic	Recommendation	Reported on (page/section)
15*	**Outcome Data**	Cohort study—Report numbers of outcome events or summary measures over time.	Sections 3.2–3.6 + Tables 2–6: full outcome data presented including peak NRS, NRS trajectory, opioid consumption, complications, QoL/HDSS changes, satisfaction, multivariable predictors.
16(a)	**Main Results — Estimates**	Give unadjusted estimates and, if applicable, confounder-adjusted estimates and their precision (e.g., 95% confidence interval). Make clear which confounders were adjusted for and why they were included.	Tables 2–5 (unadjusted between-group comparisons); Table 6 + Figure 3 (multivariable adjusted odds ratios with 95% CIs for moderate-to-severe pain, adjusted for age, sex, BMI, body regions, baseline HDSS, analgesic group).
16(b)	**Main Results — Categorization**	Report category boundaries when continuous variables were categorized.	Section 2.4 (HDSS severity dichotomization: Grade 1-2 vs 3-4); Section 2.5 (NRS ≥ 4 = moderate-to-severe pain threshold based on Farrar et al. [39]).
16(c)	**Main Results — Translate Estimates**	If relevant, consider translating estimates of relative risk into absolute risk for a meaningful time period.	Section 3.2: 38% relative reduction in MME, 24-percentage-point absolute reduction in NRS ≥ 4 (74% → 42%), 26-percentage-point reduction in rescue analgesia requirement.
17	**Other Analyses**	Report other analyses done—e.g., analyses of subgroups and interactions, and sensitivity analyses.	Section 3.7 + Table 7 (sensitivity analysis by enrollment year); Supplementary Table S2 (FDR-adjusted p-values); Section 4 (subgroup discussion: gender, HDSS severity).
		Discussion	
18	**Key Results**	Summarise key results with reference to study objectives.	Section 4, opening paragraphs: 38% MME reduction, 69% reduction in adjusted odds of moderate-to-severe pain, accelerated functional recovery.
19	**Limitations**	Discuss limitations of the study, taking into account sources of potential bias or imprecision. Discuss both direction and magnitude of any potential bias.	Section 4.1 (Limitations): six explicit limitations — non-randomized design with allocation bias, lack of blinding, opioid-only control without multimodal analgesia, single-center design, short follow-up window, multiple comparisons (FDR-corrected).
20	**Interpretation**	Give a cautious overall interpretation of results considering objectives, limitations, multiplicity of analyses, results from similar studies, and other relevant evidence.	Section 4: comparison with prior literature (Stamenkovic, Han, Yoo, Minqiang, etc.); discussion of findings relative to ERAS recommendations; explicit statement that confirmation in RCT is needed.
21	**Generalisability**	Discuss the generalisability (external validity) of the study results.	Section 4.1 (Limitations): generalizability limited by single-center design, specific institutional protocols (semi-Fowler position, harmonic scalpel, T3-T4 transection), and population characteristics; multicenter validation recommended.
		Other Information	
22	**Funding**	Give the source of funding and the role of the funders for the present study and, if applicable, for the original study on which the present article is based.	Section 'Funding': 'This research received no external funding.'

References cited in the checklist:

[33] Centers for Disease Control and Prevention. Calculating Total Daily Dose of Opioids for Safer Dosage. Atlanta, GA: U.S. Department of Health and Human Services, 2017.

[34] Farrar JT, Young JP, LaMoreaux L, Werth JL, Poole RM. Clinical importance of changes in chronic pain intensity measured on an 11-point numerical pain rating scale. Pain 2001;94:149–158.

Original STROBE reference: *von Elm E, Altman DG, Egger M, Pocock SJ, Gøtzsche PC, Vandenbroucke JP; STROBE Initiative. The Strengthening the Reporting of Observational Studies in Epidemiology (STROBE) statement: guidelines for reporting observational studies. Lancet 2007;370(9596):1453–1457. doi:10.1016/S0140-6736(07)61602-X.*
