# Supplementary material for: Intercostal Nerve Block for Bilateral Thoracoscopic Sympathectomy: A Prospective Observational Cohort Comparison of Three Analgesic Protocols
Source: J Clin Med. 2026 Jul 22;15(14):5755. doi: 10.3390/jcm15145755 (PMC13412612; doi:10.3390/jcm15145755)
Supplement: Supplementary file 1 [file jcm-15-05755-s001.zip › Supplementary tables S1 and S2.pdf]

Supplementary Table S1. Distribution of Group Allocation by Enrollment Month

Enrollment Month	Group 1 (Opioid only)	Group 2 (LA infiltration)	Group 3 (ICNB)	Monthly total, n	ICNB share (%)	Cumulative n (running total)
			Year 2018			
Feb 2018	5	4	**1**	10	10%	*10*
Mar 2018	4	4	**2**	10	20%	*20*
Apr 2018	4	3	**2**	9	22%	*29*
May 2018	4	4	**2**	10	20%	*39*
Jun 2018	4	3	**3**	10	30%	*49*
Jul 2018	3	3	**2**	8	25%	*57*
Aug 2018	3	3	**3**	9	33%	*66*
Sep 2018	3	3	**3**	9	33%	*75*
Oct 2018	3	2	**3**	8	38%	*83*
Nov 2018	3	3	**3**	9	33%	*92*
Dec 2018	3	2	**3**	8	38%	*100*
**2018 subtotal**	**39**	**34**	**27**	**100**	**27%**	—
			Year 2019			
Jan 2019	3	3	**2**	8	25%	*108*
Feb 2019	3	3	**3**	9	33%	*117*
Mar 2019	3	3	**3**	9	33%	*126*
Apr 2019	3	3	**3**	9	33%	*135*
May 2019	3	3	**3**	9	33%	*144*
Jun 2019	3	3	**3**	9	33%	*153*
Jul 2019	3	3	**3**	9	33%	*162*
Aug 2019	3	3	**3**	9	33%	*171*
Sep 2019	3	3	**3**	9	33%	*180*
Oct 2019	2	2	**3**	7	43%	*187*
Nov 2019	3	2	**2**	7	29%	*194*
Dec 2019	2	2	**2**	6	33%	*200*
**2019 subtotal**	**34**	**33**	**33**	**100**	**33%**	—
			Year 2020			
Jan 2020	3	3	**3**	9	33%	*209*
Feb 2020	3	3	**3**	9	33%	*218*
Mar 2020	2	3	**3**	8	38%	*226*
Apr 2020	2	3	**3**	8	38%	*234*
May 2020	2	3	**3**	8	38%	*242*
Jun 2020	2	3	**4**	9	44%	*251*
Jul 2020	2	3	**4**	9	44%	*260*
Aug 2020	2	2	**4**	8	50%	*268*
Sep 2020	3	3	**4**	10	40%	*278*
Oct 2020	2	3	**4**	9	44%	*287*
Nov 2020	2	2	**3**	7	43%	*294*
Dec 2020	2	2	**2**	6	33%	*300*
**2020 subtotal**	**27**	**33**	**40**	**100**	**40%**	—
**OVERALL TOTAL**	**100**	**100**	**100**	**300**	**33%**	**300**

Footnote. *Numbers represent the count of patients enrolled in each group during each calendar month. The 'ICNB share' column shows the percentage of monthly enrollments allocated to Group 3 (intercostal nerve block); this percentage rose from approximately 13–18% during the first months of 2018 to 30–40% during 2020, reflecting institutional uptake of the technique as anesthesiologists gained familiarity. Year subtotals match the totals reported in Table 7 of the main manuscript (2018: G1=39, G2=34, G3=27; 2019: G1=34, G2=33, G3=33; 2020: G1=27, G2=33, G3=40). The overall ICNB share across the entire enrollment period was 100/300 = 33.3%.Statistical implication: The non-uniform monthly distribution does not affect the validity of within-group analyses but does support the rationale for the sensitivity analysis stratified by enrollment year (Table 7). After adjustment for enrollment year as a categorical fixed effect in the linear mixed-effects model, the*

protective association of ICNB with peak NRS was preserved (β = −1.41, p < 0.001), with no significant main effect of enrollment year (p = 0.872), indicating that the temporal trend in ICNB uptake does not account for the observed between-group differences in pain outcomes.

Supplementary Table S2. Raw and Benjamini-Hochberg FDR-adjusted p-values for secondary outcomes

Outcome (Table)	Comparison	Raw p	FDR-adjusted p	Significant after FDR?
NRS at 6 h (Table 2)	Overall 3-group	<0.001	<0.001	Yes
NRS at 12 h (Table 2)	Overall 3-group	<0.001	<0.001	Yes
NRS at 24 h (Table 2)	Overall 3-group	<0.001	<0.001	Yes
Moderate-to-severe pain (Table 2)	Overall 3-group	<0.001	<0.001	Yes
Pain duration trend (Table 2)	Cochran-Armitage	0.004	0.011	Yes
Rescue analgesia required (Table 2)	Overall 3-group	<0.001	<0.001	Yes
Port-site NRS (Table 3)	Overall 3-group	<0.001	<0.001	Yes
Anterior chest wall NRS (Table 3)	Overall 3-group	0.004	0.011	Yes
Posterior thorax NRS (Table 3)	Overall 3-group	<0.001	<0.001	Yes
PONV (Table 4)	Overall 3-group	0.194	0.272	No
Length of stay (Table 4)	Overall 3-group	0.002	0.007	Yes
Return to normal activity (Table 5)	Kruskal-Wallis	0.008	0.019	Yes
Very satisfied (Table 5)	Overall 3-group	0.044	0.077	No (borderline)
Female gender (Table 6)	AOR vs. 1.0	0.046	0.077	No (borderline)
Severe HDSS (Table 6)	AOR vs. 1.0	0.021	0.044	Yes

Benjamini-Hochberg false discovery rate (FDR) correction was applied across the family of 15 secondary outcome p-values listed above. Primary outcomes (peak NRS, total opioid consumption, ICNB AOR in multivariable model) are not included in the correction family because they were pre-specified as primary endpoints. The 'very satisfied' (p = 0.044 → 0.077) and 'female gender' (p = 0.046 → 0.077) findings lose significance after FDR correction and are interpreted as hypothesis-generating in the Discussion. AOR, Adjusted Odds Ratio; FDR, False Discovery Rate; HDSS, Hyperhidrosis Disease Severity Scale; NRS, Numerical Rating Scale; PONV, Postoperative Nausea and Vomiting.
